# The Temporal Order of DNA Replication Shaped by Mammalian DNA Methyltransferases

**DOI:** 10.3390/cells10020266

**Published:** 2021-01-29

**Authors:** Shin-ichiro Takebayashi, Tyrone Ryba, Kelsey Wimbish, Takuya Hayakawa, Morito Sakaue, Kenji Kuriya, Saori Takahashi, Shin Ogata, Ichiro Hiratani, Katsuzumi Okumura, Masaki Okano, Masato Ogata

**Affiliations:** 1 Laboratory of Molecular and Cellular Biology, Graduate School of Bioresources, Mie University, Tsu, Mie 514-8507, Japan; 519d303@m.mie-u.ac.jp (T.H.); kuriya@bio.mie-u.ac.jp (K.K.); shin@bio.mie-u.ac.jp (S.O.); katsu@bio.mie-u.ac.jp (K.O.); 2 Division of Natural Sciences, New College of Florida, Sarasota, FL 34243, USA; tryba@ncf.edu (T.R.); kelsey.wimbish13@ncf.edu (K.W.); 3 Department of Orthopaedic Surgery, Osaka University Graduate School of Medicine, Suita, Osaka 565-0871, Japan; moritonism@gmail.com; 4 Laboratory for Developmental Epigenetics, RIKEN BDR, Kobe, Hyogo 650-0047, Japan; saori.takahashi@riken.jp (S.T.); ichiro.hiratani@riken.jp (I.H.); 5 Institute of Molecular Embryology and Genetics, Kumamoto University, Kumamoto 860-0811, Japan; okano@kumamoto-u.ac.jp; 6 Department of Biochemistry and Proteomics, Graduate School of Medicine, Mie University, Tsu, Mie 514-8507, Japan; ogata@doc.medic.mie-u.ac.jp

**Keywords:** DNA replication, replication timing, DNA methyltransferases

## Abstract

Multiple epigenetic pathways underlie the temporal order of DNA replication (replication timing) in the contexts of development and disease. DNA methylation by DNA methyltransferases (Dnmts) and downstream chromatin reorganization and transcriptional changes are thought to impact DNA replication, yet this remains to be comprehensively tested. Using cell-based and genome-wide approaches to measure replication timing, we identified a number of genomic regions undergoing subtle but reproducible replication timing changes in various *Dnmt*-mutant mouse embryonic stem (ES) cell lines that included a cell line with a drug-inducible Dnmt3a2 expression system. Replication timing within pericentromeric heterochromatin (PH) was shown to be correlated with redistribution of H3K27me3 induced by DNA hypomethylation: Later replicating PH coincided with H3K27me3-enriched regions. In contrast, this relationship with H3K27me3 was not evident within chromosomal arm regions undergoing either early-to-late (EtoL) or late-to-early (LtoE) switching of replication timing upon loss of the Dnmts. Interestingly, Dnmt-sensitive transcriptional up- and downregulation frequently coincided with earlier and later shifts in replication timing of the chromosomal arm regions, respectively. Our study revealed the previously unrecognized complex and diverse effects of the Dnmts loss on the mammalian DNA replication landscape.

## 1. Introduction

Mammalian chromosomes consist of multiple replication origins. Coordinate activation of multiple origins across the scale of several hundred kilobases to several megabases forms replication domains, the regulatory units of DNA replication [1,2,3]. Genome-wide replication timing analysis has revealed the periodic distribution of early and late replication domains along the chromosome arms [4]. Replication domains are reorganized in the contexts of development and disease, giving rise to cell type-specific organization of replication domains [4]. For example, the chromosomal region that contains pluripotency-associated genes such as *Dppa2*/*Dppa4* displays early replication in mouse ES cells, while in closely related epiblast stem cells, the same region switches to late replication [5]. Replication timing of some genomic regions is commonly dysregulated in pediatric leukemia patient cells [6]. Given these observations, epigenetic mechanisms are thought to play important roles in the temporal regulation of DNA replication. To date, however, only a handful of epigenetic modifiers have been tested to determine whether and to what extent they contribute towards the DNA replication program [7].

DNA methylation is an epigenetic modification found in gene regulatory regions, heterochromatin, DNA repeats, and transposable elements [8,9]. DNA methylation patterns in the genome are stably inherited over many cell divisions by the cooperative action of the DNA methyltransferases (Dnmts) [10,11]. Regulation of gene transcription by DNA methylation has been well studied. Mechanistically, methylated CpGs are recognized by a set of methyl CpG-binding proteins (MBDs and the BTB/POZ domain family of proteins) that recruit chromatin-modifying complexes and bring about repressive chromatin environments near gene promoter regions [12]. Mouse pericentromeric heterochromatin (PH) forms a microscopically observable nuclear domain that contains highly methylated DNA [13]. Not only Dnmts, but also MDBs are localized at PH, thus providing a model to examine the relationship between DNA methylation and histone modifications [14,15]. Genetic inactivation of genes responsible for DNA methylation leads to aberrant gene expression patterns [16,17,18]. Several biochemical studies have shown the interaction of DNA methyltransferases with various histone modifiers, suggesting that non-catalytic functions of Dnmts also contribute to the regulation of gene expression [19,20]. However, the roles of the Dnmts other than gene regulation are poorly understood. DNA hypomethylation is associated with the promotion of tumorigenesis and other disease states [21,22], and a causal relationship between DNA hypomethylation and chromosome instability has been reported; however, the underlying mechanisms remain unclear [23,24,25].

Although genome-wide replication timing analysis has not been performed in Dnmt-deficient cells, a global correlation between DNA methylation and early replication has been observed [26]. This is somewhat unexpected since the Dnmts and DNA methylation generally induce repressive chromatin environments that favor late replication. To directly examine the extent to which the loss of Dnmts and DNA methylation affect the DNA replication program, we performed detailed replication timing analysis using genetically engineered *Dnmt*-mutant mouse ES cell lines.

## 2. Materials and Methods

### 2.1. Cell Culture

*Dnmt1^−/−^Dnmt3a^−/−^Dnmt3b^−/−^* triple-knockout (TKO) ES cells (clone 19-1) [27] and wild-type (WT) J1 ES cells were maintained on gelatin-coated culture dishes in KnockOut Dulbecco’s Modified Eagle Medium (Thermo Fisher Scientific, Waltham, MA, USA) supplemented with 7.5% (*v/v*) ES-qualified FBS (Hyclone, Logan, UT, USA), 7.5% (*v/v*) KnockOut Serum Replacement (Thermo Fisher Scientific, Waltham, MA, USA), 2 mM L-glutamine, 10 mM HEPES, 100 U/mL penicillin/streptomycin, 0.1 mM non-essential amino acids, and 0.1 mM β-mercaptoethanol in the presence of leukemia inhibitory factor (LIF) at 37 °C with 5% CO_2_.

*Dnmt3a^−/−^Dnmt3b^−/−^* double-knockout (DKO) ES cell clones stably expressing WT or catalytic-defective (C487S) Dnmt3a2 were generated by electroporation-mediated introduction of cDNA expression plasmids driven by the CAG promoter, followed by selection with blasticidin. An empty expression plasmid was used to generate control mock clones.

*Dnmt3a^−/−^Dnmt3b^−/−^* DKO ES cell clones stably expressing RU486 (mifepristone, a progesterone receptor antagonist)-inducible Dnmt3a2 were generated by electroporation-mediated introduction of an expression plasmid for Dnmt3a2 fused with a modified ligand-binding domain of the human progesterone receptor (termed “GPRh”) driven by the CAG promoter, followed by selection with blasticidin. A mammalian expression vector with the RU486-inducible GPRh was provided by Hitoshi Niwa, Kumamoto University. The GPRh is a chimeric protein comprising the human glucocorticoid receptor (513-554; UniProt ID, P04150) and the human progesterone receptor (710-914; UniProt ID, P06401), in which the N-terminal helices 1 and 2 in the ligand-binding domain of the progesterone receptor are replaced with those of the glucocorticoid receptor to increase the capacity for cytosolic retention in the absence of ligand [28], and the C-terminal 19 amino acids of the progesterone receptor are deleted allowing response to RU486 but not to progesterone [29,30]. Five amino acids (MHAYG) derived from the cloning site are added at the C-terminal end. To induce expression of the Dnmt3a2 protein, ES cells (clone 1723) were cultured in ES medium containing 1 µM RU486 for 14 days on feeder mouse embryonic fibroblast (MEF) cells. Control cells were treated with ethanol only.

### 2.2. Genome-Wide Replication Timing Analysis

Replication timing analysis with BrdU labeling was performed as described previously [31,32,33] with modifications. In brief, cells were labeled with 50 µM BrdU for 2 h, washed twice with ice-cold PBS, trypsinized, and then fixed in 75% (*v/v*) ethanol. These cells were resuspended in PBS containing 1% FBS, stained with propidium iodide (20 µg/mL) for 30 min in the presence of RNase A (250 µg/mL), and then sorted into early and late S phase fractions by flow cytometry (SH800 Cell Sorter; Sony Biotechnology, Tokyo, Japan). After phenol-chloroform extraction of the cellular DNA, immunoprecipitation with anti-BrdU mouse monoclonal antibody (Cat#555627; BD Biosciences, San Jose, CA, USA) was performed for each fraction to enrich BrdU-substituted replicating DNA. Isolated early and late replicating DNA were amplified by whole-genome amplification (WGA) (SeqPlex Enhanced DNA Amplification Kit; Sigma-Aldrich, St. Louis, MO, USA) and used to construct libraries for sequencing with an Ion Proton next generation sequencer (Life Technologies, Waltham, MA, USA). Library preparation and data acquisition were performed according to standard Ion Proton procedures. Data analyses were performed with SeqMonk (http://www.bioinformatics.babraham.ac.uk/projects/seqmonk/) and R/Bioconductor (http://www.r-project.org; http://www.bioconductor.org). The number of total reads in each sample were normalized per million reads in SeqMonk. The ratio of sequence reads between early and late S phase samples was determined in windows of 5 kb and LOESS-smoothed over a 300-kb window size in R/Bioconductor. These smoothed datasets were used to generate the replication timing profiles.

For some analyses, datasets were averaged into 200-kb windows (fixed position) and the replication timing differential (i.e., TKO ratio − WT ratio) was determined for each 200-kb segment. To determine the significant replication timing switching domains that were independent of changes between replicates, we calculated the Euclidian distance at 13,222 200-kb segments between groups (i.e., WT vs. TKO) and within groups (i.e., WT replicate-1 vs. WT replicate-2), which was used to calculate the *p*-values at each 200-kb genomic segment [34]. Statistical significance was then calculated using the qvalue package in R/Bioconductor, which yields a *q*-value for each segment that reflects the proportion of false positives (i.e., the false discovery rate; FDR) among segments deemed to have significant replication timing changes. High-confidence replication timing switching domains were selected with a *q*-value cutoff of 0.01, corresponding to an overall FDR of 1%. A *q*-value cutoff of 0.05 was also used to identify a set of lower-confidence domains. 

### 2.3. DNA Combing

DNA combing and immunofluorescence detection of replication-labeled DNA were performed as described previously [35]. Cells were first labeled with 100 µM 5-iodo-2′-deoxyuridine (IdU; Sigma-Aldrich, St. Louis, MO, USA) for 20 min. After washing with PBS, the cells were labeled with 100 µM 5-chloro-2′-deoxyuridine (CldU; Sigma-Aldrich, St. Louis, MO, USA) for 20 min. The labeling reaction was stopped by washing the cells with ice-cold PBS. The cells were collected by centrifugation at 400× *g* for 3 min at 4 °C, and the supernatant was removed before adding buffer A (250 mM sucrose, 20 mM HEPES, pH 7.0, 10 mM KCl, 1.5 mM MgCl_2_, 1 mM EDTA, 1 mM EGTA) to the cell pellets. Then, agarose LM-MP (Roche, Mannheim, Germany) was added to a final concentration of 0.7% (*w/v*), and the resultant cell suspension was transferred into a plug mold. After 2 h of incubation on ice, the solidified gel containing the cells was incubated in ESP solution (10 mM N-lauroylsarcosine sodium salt, 10 mM Tris-HCl, pH 8.0, 0.5 M EDTA, 2 mg/mL Proteinase K) for 16 h at 50 °C to digest the proteins. Next, the gel was washed with 0.5 M EDTA three times and then washed twice with 1× TE (10 mM Tris-HCl, pH 8.0, 1 mM EDTA). After 1 h of incubation at room temperature, the gel was washed with 1× TE and incubated with 50× β-agarase buffer (Lonza Japan, Tokyo, Japan) for 1 h. Then, the gel was melted at 70 °C, and β-agarase I (Lonza Japan, Tokyo, Japan) was added to complete the gel dissolution. Finally, the DNA was diluted with 0.5 M MES, pH 5.5 and stored at 4 °C until use. DNA molecules isolated in the MES buffer were combed at a rate of 300 µm/s onto silanated coverslips (Matsunami Glass, Osaka, Japan) as described previously [36]. To estimate the degree of DNA molecule stretching on the coverslips, λ-DNA molecules were similarly combed and stained with YOYO-1 for length measurement. Because the λ phage virus genome is 48.5 kb in length, the rate of DNA stretching was calculated as 2.32 ± 0.11 kb/mm. Combed DNA molecules were denatured in 50% formamide and 2× SSC (saline-sodium citrate) buffer for 12 min at 72 °C. Denatured DNA molecules were incubated in blocking solution (3% Blocking Reagent from Sigma-Aldrich, and 0.05% Tween 20 in PBS) for 30 min at 37 °C to reduce nonspecific binding, followed by incubation in a detection solution (1% Blocking Reagent, 0.05% Tween 20 in PBS) containing the primary antibodies for 1 h at 37 °C. A mouse anti-BrdU monoclonal antibody (1:5 dilution, clone B44, mouse IgG1, Cat#347580; BD Biosciences, San Jose, CA, USA) and rat anti-BrdU monoclonal antibody (1:50 dilution, clone BU1/75, rat IgG2a, Cat#ab6326; Abcam, Cambridge, UK) were used for immunodetection of IdU- and CldU-labeled DNA, respectively. After washing with 0.05% Tween 20/PBS, the DNA molecules were incubated in detection solution containing Alexa Fluor 555-conjugated goat anti-mouse IgG (1:100 dilution; Life Technologies, Carlsbad, CA, USA) and Alexa Fluor 488-conjugated rabbit anti-rat IgG (1:100 dilution; Life Technologies, Carlsbad, CA, USA) for 30 min at 37 °C. After washing with 0.05% Tween 20/PBS, the coverslips were mounted in Vectashield (Vector Laboratories, Burlingame, CA, USA).

### 2.4. Immunofluorescence Staining

ES cells grown on culture dishes were collected by trypsinization, cytospun onto glass slides for 10 min at 1350 rpm, fixed with 4% paraformaldehyde in PBS (10 min, 25 °C), washed, and then permeabilized with 0.5% Triton X-100 in PBS (10 min, 25 °C). For immunostaining, the samples were incubated in blocking solution (3% BSA, 0.1% Tween 20 in 4× SSC) for 30 min at 37 °C to reduce nonspecific binding, and then in detection solution (1% BSA, 0.1% Tween 20 in 4× SSC) containing primary antibodies for 1 h at 37 °C. After three washes with 4× SSC, the samples were incubated in detection solution containing the secondary antibodies. The primary antibodies were: anti-PCNA mouse monoclonal antibody (1:20 dilution, Cat#sc-56; Santa Cruz Biotechnology, Santa Cruz, CA, USA), anti-H3K27me3 mouse monoclonal antibody (1:200 dilution, Cat#05-851; Millipore, Billerica, MA, USA), anti-H3K27me3 rabbit polyclonal antibody (1:200 dilution, Cat#07-449; Millipore, Billerica, MA, USA), anti-H3K9me3 rabbit polyclonal antibody (1:500 dilution, Cat#07-442; Millipore, Billerica, MA, USA), anti-H4K20me3 rabbit polyclonal antibody (1:500 dilution, Cat#07-463; Millipore, Billerica, MA, USA), anti-phospho-H3S10 rabbit polyclonal antibody (1:100 dilution, Cat#06-570; Millipore, Billerica, MA, USA), anti-AIM-1/Aurora-B mouse monoclonal antibody (1:50 dilution, Cat#611082; BD Biosciences, Bedford MA, USA), anti-HP1β mouse monoclonal antibody (1:500 dilution, Cat# MAB3448; Chemicon International, Hofheim, Germany). Alexa Fluor 488 goat anti-mouse IgG (Cat# A11017; Molecular Probes, Carlsbad, CA, USA), Alexa Fluor 555 goat anti-rabbit IgG (Cat#A21430; Molecular Probes, Carlsbad, CA, USA), and Alexa Fluor 647 goat anti-rabbit IgG (Cat#A21245; Molecular Probes, Carlsbad, CA, USA) were used as secondary antibodies. Before imaging, the slides were counterstained with 4′,6-diamidino-2-phenylindole (DAPI, 200 ng/mL; Life Technologies, Carlsbad, CA, USA), washed with 4× SSC and then mounted in 90% glycerol containing an antifade reagent.

### 2.5. Visualization of Replication Sites in the Cell Nucleus

**Figure 1:**
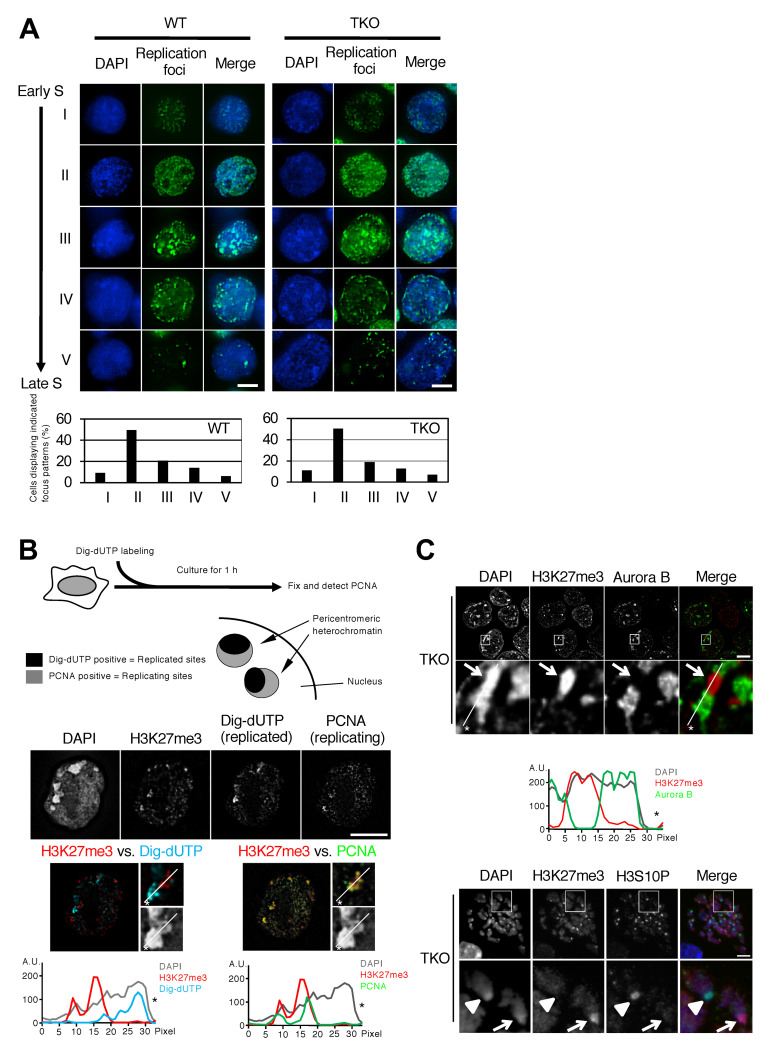
H3K27me3 foci formed in mouse embryonic stem cells with severely hypomethylated DNA coincide with later replication of pericentromeric heterochromatin. (**A**) Spatial regulation of DNA replication sites in *Dnmt1^−/−^Dnmt3a^−/−^Dnmt3b^−/−^* triple-knockout (TKO) mouse embryonic stem (ES) cell nuclei during S phase. Asynchronously growing cells were labeled with EdU (green) for 10 min to visualize sites of DNA synthesis in the nucleus. Five representative images of the distribution patterns of DNA replication foci during S phase over time (I–V) are shown [40,41]. Nuclear DNA was stained with DAPI (blue). The percentage of cells displaying each focus pattern was scored (*n* > 200). (**B**) The top panel shows the experimental strategy used to determine the temporal order of replication within pericentromeric heterochromatin (PH) regions of TKO ES cells where abnormal H3K27me3 foci were formed. Unsynchronized cells were first labeled with DIG-dUTP, cultured for 1 h, and then fixed for PCNA/H3K27me3 immunostaining. Both DIG-dUTP-positive and PCNA-positive PH were selected to measure colocalization with H3K27me3 foci. During the 1 h culture period, cells proceeded to subsequent stages of S phase, which allowed us to spatially distinguish already replicated sites (DIG-dUTP-labeled) from currently replicating sites (PCNA-labeled) within PH regions (middle panel). H3K27me3 foci colocalized only with PCNA-positive, later replicating PH, producing the yellow color in the merged image (bottom right). The charts in the bottom panel show the immunofluorescence signal intensities for each label (A.U., arbitrary units) plotted along the white line shown in the magnified immunofluorescence images of the PH regions. H3K27me3 and DIG-dUTP exhibited a mutually exclusive distribution in DAPI-dense heterochromatin regions. However, H3K27me3 distribution coincided with that of PCNA. Asterisks in the images indicate the direction of line scanning. (**C**) Perturbed Aurora B recruitment and loss of histone H3 phosphorylation at H3K27me foci were observed in the interphase nuclei of the TKO ES cells (top panel). Arrows in the magnified views indicate the H3K27me3-enriched PH regions where Aurora B signals were depleted. The chart (middle panel) shows the intensities of each immunofluorescence signal as in (**B**). Asterisks in the images indicate the direction of line scanning. Immunofluorescence detection of phosphorylated histone H3 at Ser10 (H3S10P) together with H3K27me3 on mitotic chromosomes is also shown (bottom panel). Intense H3S10P signals were not detected in the H3K27me3-enriched PH of TKO ES cells. Arrows in the magnified views indicate the H3K27me3-enriched PH of a mitotic chromosome where H3S10P signals were depleted, while the arrowheads indicate the non-H3K27me3-enriched PH where the accumulation of H3S10P normally occurs. Cell images were collected at multiple stage positions using a deconvolution fluorescence microscope (see details in Materials and Methods) and images at one focal plane are shown. Scale bars, 10 µm.

Cells were labeled with 100 µM EdU (Life Technologies, Carlsbad, CA, USA) for 10 min, aliquoted onto coverslips and fixed with 4% paraformaldehyde in PBS, and then permeabilized with 0.5% Triton X-100 in PBS. Blocking was performed by incubation in 3% BSA/0.1% Tween 20/Tris-buffered saline, pH for 30 min. EdU-labeled DNA was detected with the Click-iT EdU Alexa Fluor 488 Imaging Kit (Life Technologies, Carlsbad, CA, USA) according to the manufacturer’s protocol. Digoxigenin (DIG)-labeled DNA was detected with an anti-DIG antibody conjugated with FITC (1:200 dilution; Roche, Mannheim, Germany). DAPI was used to counterstain the DNA. In the experiment in Figure 1B, digoxigenin-11-dUTP (DIG-dUTP; Roche, Mannheim, Germany) was loaded into cells with the hypotonic shift method for replication labeling [37]. Incorporated DIG-dUTP was detected with an anti-DIG antibody conjugated with rhodamine (Cat#11207750910; Roche, Mannheim, Germany).

### 2.6. Immuno-DNA FISH

Cells cytospun onto glass slides were fixed with 4% paraformaldehyde for 10 min, washed, and then permeabilized with 0.5% Triton X-100 in PBS. After immunostaining with the H3K27me3 antibody as described above, samples were refixed with 4% paraformaldehyde for 10 min and then heat denatured in 50% formamide and 2× SSC for 10 min at 80 °C. Denatured samples were immersed in 70% ethanol for 5 min at −20 °C and dehydrated with a 90–100% ethanol gradient. To generate DNA FISH probes, major satellite, minor satellite, and telomere genomic DNA fragments amplified by PCR were labeled by nick translation. The following primer sets were used: Major satellite-f (5′-AGTGTGCCGTTGTCTCTTCG-3′), major satellite-r (5′-ACACCAGAGTGCAAGACAGC-3′), minor satellite-f (5′-AGAACATATTAGATGAGT-3′), minor satellite-r (5′-ACTCATCTAATATGTTCT-3′), telomere-f (5′-TTAGGGTTAGGGTTAGGGTTAGGGTTAGGG-3′), and telomere-r (5′-CCCTAACCCTAACCCTAACCCTAACCCTAA-3′). Cells were treated with 0.5% Triton X-100 in CSK buffer (10 mM PIPES, pH 6.8, 100 mM NaCl, 300 mM sucrose, 3 mM MgCl_2_, 1 mM EGTA) for 30 s at 4 °C, fixed with 4% paraformaldehyde, and then immersed in 70% ethanol for 5 min at −20 °C. The cells were dehydrated with a 90–100% ethanol gradient, and the denatured FISH probe mixture was hybridized to slides at 37 °C for 16 h in a moist chamber. Slides were washed three times with 50% formamide in 2× SSC at 43 °C and then washed a further three times with 0.8× SSC at 60 °C. Slides were incubated for 30 min in a blocking solution (3% BSA, 0.1% Tween 20 in 2× SSC) at 37 °C and incubated in a detection solution (1% BSA, 0.1% Tween 20 in 2× SSC) containing anti-DIG-conjugated rhodamine for 30 min at 37 °C. Then, slides were washed three times with 4× SSC/0.1% Tween 20 for 5 min at 43 °C. Before imaging, the slides were counterstained with DAPI (200 ng/mL), washed with 4× SSC, and then mounted in 90% glycerol containing an antifade reagent.

### 2.7. Microarray Analysis

Total cellular RNA from two independent experiments was reverse transcribed and hybridized to the Affymetrix Mouse Genome 430 2.0 microarray, as described previously [38]. Data processing, quality control, and output file generation was performed with Affymetrix Gene Expression Console. Raw data (CEL files) were used to perform normalization through the Robust Multiarray Analysis (RMA) algorithm.

### 2.8. Methylation Analysis by Southern Blotting

Genomic DNA was digested with CpG methylation-sensitive restriction enzymes (HpaII or MaeII), blotted, and hybridized with major satellite (pSAT) probes as described previously [39].

### 2.9. Imaging System and Measurements

Images were collected using a Leica DM RA2 fluorescence microscope equipped with a cooled charge-coupled device camera (C4742-95-12ER; Hamamatsu Photonics, Shizuoka, Japan), controlled by an Apple Macintosh G4 computer running the software program IPLab (Signals Analytics, Vienna, VA, USA). The images were captured at different stage positions and processed with the Huygens Essential deconvolution software (Scientific Volume Imaging, Hilversum, Netherlands). For some experiments, a fluorescence microscope (Axioplan 2 MOT; Carl Zeiss, Jena, Germany) equipped with an ORCA R2 camera (Hamamatsu Photonics, Shizuoka, Japan) was used for imaging.

## 3. Results

### 3.1. H3K27me3 Foci Formed in Mouse Embryonic Stem Cells with Severely Hypomethylated DNA Coincide with Later Replication of Pericentromeric Heterochromatin

Intranuclear distribution patterns of DNA replication sites are known to change dynamically during S phase: DNA replication initially occurs throughout the interior part of the nucleus, then in the region of the pericentromeric heterochromatin (PH) that is densely stained with DAPI, and last in the nuclear periphery [40,41]. To examine the effect of Dnmt loss on the DNA replication program, we first investigated the spatial organization of DNA replication sites in *Dnmt1^−/−^Dnmt3a^−/−^Dnmt3b^−/−^* triple-knockout (TKO) mouse ES cells. We observed that the S phase stage-specific distribution patterns of DNA replication sites (patterns I–V) and their frequency in TKO ES cells were comparable to those in wild-type ES cells (Figure 1A), suggesting that spatial regulation of the DNA replication program is largely maintained in the absence of the Dnmts. We also did not detect a significant difference between wild-type and TKO ES cells in the rate of replication fork progression and ori-to-ori distance as measured by a DNA combing assay (Figure S1).

A previous report demonstrated that H3K27me3 foci are formed in the PH of extensively DNA hypomethylated cells [42]. Consistent with this, we observed H3K27me3 foci formation at major satellite repeats, which are a main structural component of PH (Figure S2A,B), and regional exclusion of normally enriched constitutive heterochromatin markers such as HP1β, H3K9me3, and H4K20me3 (Figure S2C,D). Although H3K27me3 foci are formed in a DNA methylation-dependent manner (Figure S3), a portion of PH in TKO ES cells still maintains the constitutive heterochromatin marks without H3K27me3 enrichment (Figure S2C,D), suggesting that loss of DNA methylation destabilizes constitutive chromatin structure and, as a consequence, indirectly induces regional H3K27me3 foci formation within PH. Interestingly, these H3K27me3-enriched PH regions replicate later than neighboring PH regions where H3K27me3 signal intensity is low (Figure 1B, colocalization of H3K27me3 foci with PCNA-positive later replicating PH but not with DIG-dUTP-positive earlier replicating PH). In H3K27me3-enriched PH regions, we also observed loss of Aurora B recruitment and H3S10 phosphorylation, which are known to coincide with replication timing delay (Figure 1C) [43]. Because the temporal order of replication within PH is closely correlated with the level of H3K27me3, it is suggested that sporadic chromatin reorganization induced by DNA methylation loss, rather than DNA methylation loss itself, affects the temporal order of DNA replication within PH.

### 3.2. Genome-Wide Replication Timing Analysis Identified a Subset of Chromosomal Regions Sensitive to Dnmt Loss

**Figure 2:**
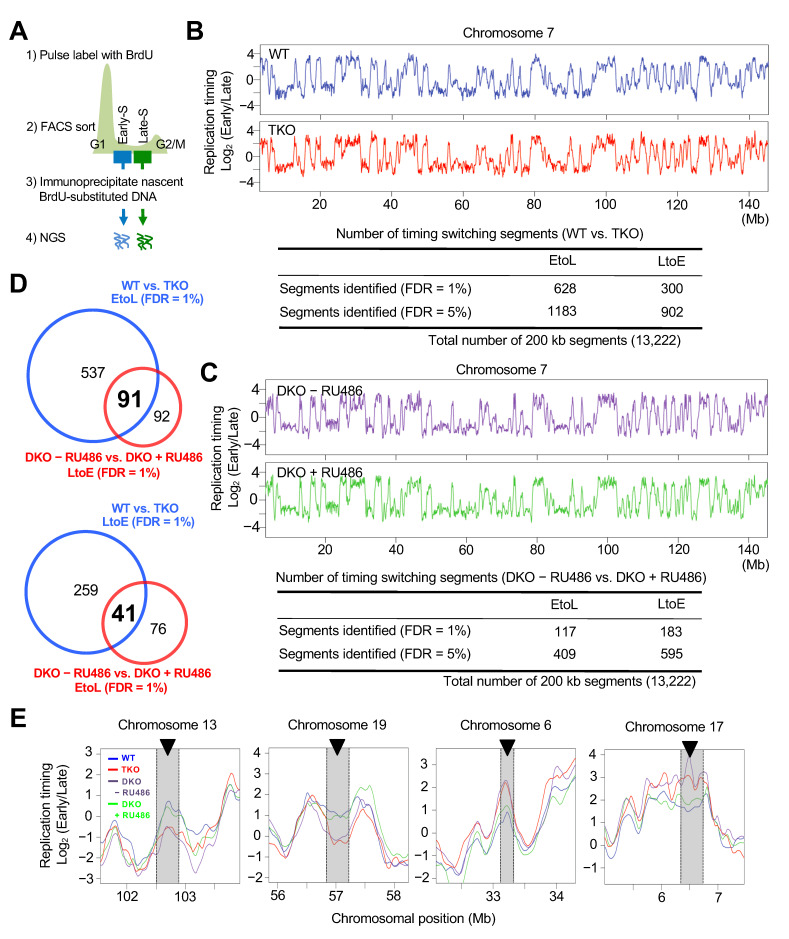
Genome-wide replication timing analysis identified a subset of chromosomal regions sensitive to Dnmt loss. (**A**) Flowchart of genome-wide replication timing analysis. BrdU-substituted DNA from early and late S phase cells was differentially labeled and sequenced by next generation sequencing. (**B**) Replication timing profiles of control wild-type and TKO ES cells for chromosome 7. The sequence read count ratio for the early and late cells (Log_2_ early/late) for each 5-kb genomic bin was plotted against the chromosomal position (top panel). The LOESS-smoothed plots for the average of two biological replicate experiments are shown. The significant EtoL and LtoE switching segments in the TKO ES cells are indicated in the table (bottom panel). Replication timing data were averaged into 200-kb windows and statistical significance was calculated between the control wild-type and TKO ES cells, as described in the Methods. (**C**) Replication timing profiles for chromosome 7 of the *Dnmt3a^−/−^Dnmt3b^−/−^* double-knockout ES cells with and without Dnmt3a2 induction (DKO + RU486 vs. DKO − RU486; top panel). The significant early-to-late (EtoL) and late-to-early (LtoE) switching segments upon Dnmt3a2 induction in the DKO ES cells are indicated in the table (bottom panel). (**D**) The overlap between genomic segments that underwent replication timing switching is shown as a Venn diagram (using the FDR = 1% data from Figure 2B,C). (**E**) Expanded plots of representative regions that underwent replication timing switches in *Dnmt*-mutant ES cells.

To systematically explore the effect of Dnmt loss on DNA replication, we next profiled the genome-wide replication timing of TKO ES cells in comparison with the parental control, wild-type ESC cells. To this end, BrdU-immunoprecipitated DNA from FACS-sorted early and late replicating fractions was analyzed using next generation sequencing (Figure 2A), and the ratio of early and late read enrichment (Log_2_ early/late) was determined against the chromosomal position (Figure 2B). Although the obtained replication timing profile of the TKO ES cells was globally similar to that of control ES cells, our detailed analysis identified a set of chromosomal regions that underwent switches from early to late (EtoL) and from late to early (LtoE) after Dnmt loss (Figure 2B). To evaluate the statistical significance of these replication timing changes, we averaged datasets into 200-kb windows (total 13,222 200-kb segments/dataset) and calculated the proportion of differences greater than those observed between biological replicates. These proportions were converted to false discovery rate (FDR; see details in Materials and Methods) estimates to account for multiple testing. Using this approach, we identified 628 and 300 200-kb segments undergoing either EtoL or LtoE switching, respectively, with an FDR of 1% FDR (Figure 2B).

To refine the genomic regions affected by the Dnmts, we also examined genome-wide replication timing in a *Dnmt3a^−/−^Dnmt3b^−/−^* double-knockout (DKO) cell line stably expressing RU486-inducible Dnmt3a2. Although we observed only partial restoration of DNA methylation upon Dnmt3a2 induction (Figure S4A), the restored DNA methylation level was sufficient to suppress H3K27me3 foci formation within the PH (Figure S4B,C). Using this inducible expression system, we found that induction of Dnmt3a2 also induced changes in replication timing in a subset of chromosomal regions (Figure 2C). As expected, regions undergoing EtoL switching upon Dnmt loss (WT vs. TKO) significantly overlapped with regions undergoing LtoE switching upon Dnmt3a2 induction (DKO − RU486 vs. DKO + RU486) (Figure 2D top). LtoE switching regions upon Dnmt loss also significantly overlapped with EtoL switching regions upon Dnmt3a2 induction, but to a lesser extent (Figure 2D bottom). These changes in replication timing were not as dramatic as changes seen in other replication domain mutants [44,45,46] but were highly reproducible between experimental replicates (Figure S5). A previous study showed that the pluripotency of ES cells is maintained in the absence of the Dnmts [27], which was further confirmed by the fact that the mutant cells used in our experiments retained pluripotent cell-specific replication timing profiles (Figure S6). Taken together, these results clearly demonstrate that the Dnmts are required to maintain the temporal order of DNA replication in a number of chromosomal arm regions in addition to PH.

### 3.3. Redistribution of the H3K27me3 Mark Is not Associated with Replication Timing Changes in the Chromosomal Arm Regions

**Figure 3:**
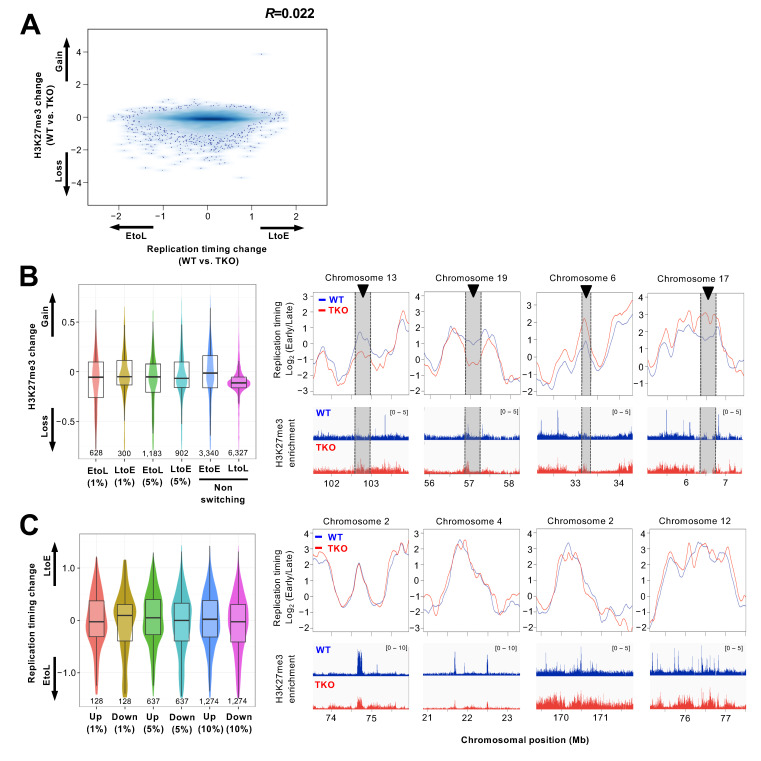
Genome-wide comparison of replication timing changes and H3K27me3 redistribution in TKO cells. (**A**) Redistribution of the H3K27me3 mark relative to changes in replication timing. Replication timing changes after Dnmt loss (TKO ESC log_2_ ratio − WT ESC log_2_ ratio) were plotted against the changes in H3K27me3 enrichment (TKO ESC − WT ESC) identified by ChIP-seq [48]. Replication timing data averaged over 200-kb segments and enrichment of H3K27me3 in each corresponding segment (12,732 segments in total) were used to calculate differences between WT and TKO. (**B**) Box plots showing the changes in H3K27me3 enrichment in EtoL and LtoE switching regions after Dnmt loss (for segments with an FDR = 1% and 5% from Figure 2C) compared with non-switching early (EtoE) or late (LtoL) regions. The number of 200-kb segments analyzed is indicated below each plot. IGV genome browser tracks illustrating the distribution of H3K27me3 within representative replication timing switching regions (from Figure 2E) are shown to the right. The scale for H3K27me3 enrichment is shown in the upper right corner. (**C**) Box plots showing the replication timing changes in genomic regions scoring in the top 1%, 5%, and 10% in terms of H3K27me3 gain and loss. The number of 200-kb segments analyzed is indicated below each plot. The replication timing profiles for representative regions undergoing redistribution of the H3K27me3 mark after Dnmt loss are shown to the right.

It has been shown that genome-wide redistribution of H3K27me3 occurs in *Dnmt* TKO ES cells [42]. In these cells, both abnormal enrichment and loss of H3K27me3 were observed in regions where this modification is normally present [42,47]. This, together with the observation that H3K27me3 enrichment coincides with later replicating PH (Figure 1B), led us to examine whether replication timing changes in the chromosomal arm regions of TKO ES cells were associated with changes in H3K27me3 enrichment. We analyzed H3K27me3 redistribution with replication timing changes using H3K27me3 ChIP-seq data from control wild-type and TKO ES cells [48] and found no appreciable correlation between the two (Figure 3A, *R* = 0.022). Genomic regions undergoing EtoL replication timing switching after Dnmt loss (regions identified in Figure 2D) exhibited no enrichment of the H3K27me3 mark but rather lost this modification, and this was also the case with the LtoE and LtoL domains (Figure 3B). Further investigation of replication timing in genomic regions that scored in the top 1%, 5%, and 10% in terms of H3K27me3 gain and loss showed poor coordination between H3K27me3 redistribution and the replication timing changes (Figure 3C). These results suggest that the association of the H3K27me3 mark with the temporal regulation of DNA replication is restricted to specific regions of the genome such as the PH and that other factors account for replication timing changes in the chromosomal arm regions.

### 3.4. Replication Timing Changes Frequently Coincide with Transcriptional Changes in the Chromosomal Arm Regions

**Figure 4:**
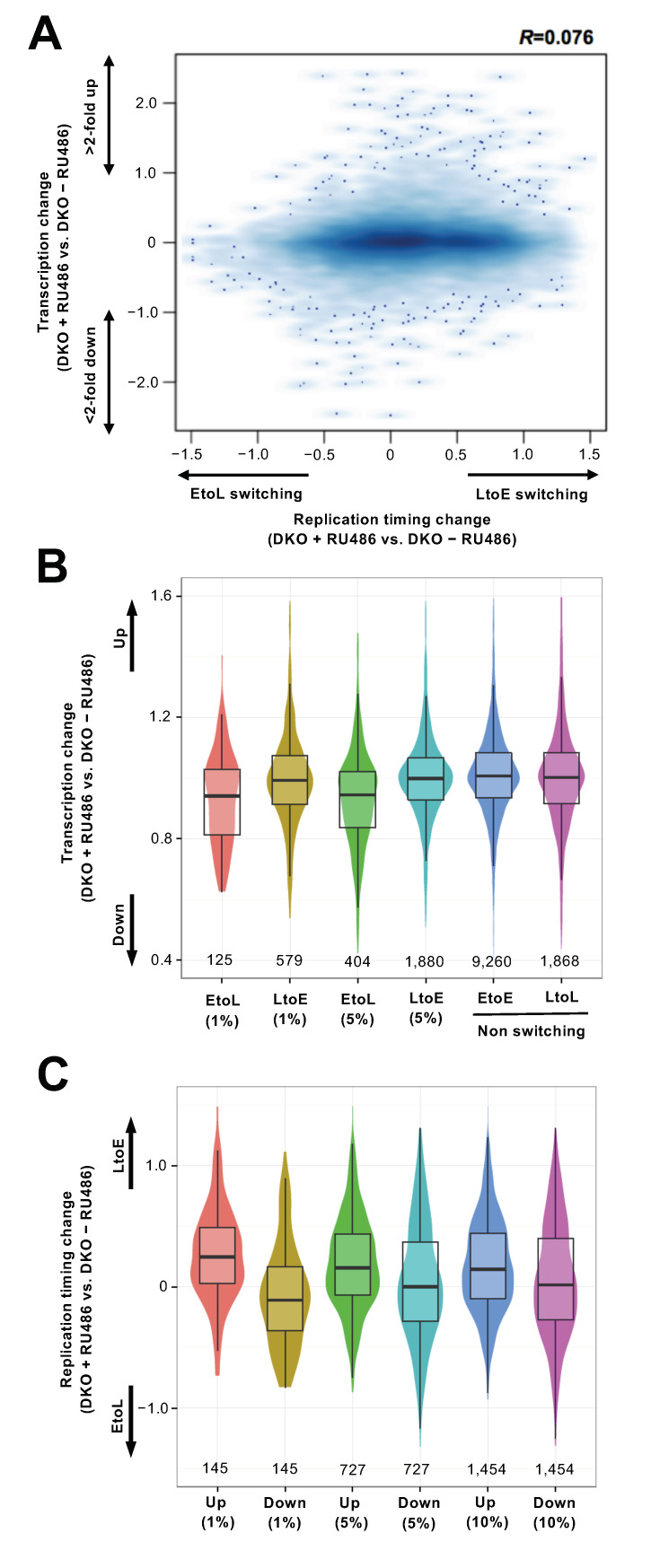
Replication timing changes upon DNA hypomethylation are associated with transcriptional changes. (**A**) Replication timing changes (DKO + RU486 log_2_ ratio − DKO − RU486 log_2_ ratio) were plotted against the transcriptional changes (log_2_(DKO + RU486/DKO − RU486)). (**B**) Box plots showing the transcriptional changes in EtoL and LtoE switching regions upon Dnmt3a2 induction (segments with an FDR = 1% and 5% from Figure 2C) compared with the non-switching early (EtoE) or late (LtoL) regions. The number of genes analyzed is indicated below each plot. The *y*-axis is the fold change (not log-transformed). (**C**) Box plots showing the replication timing changes in genomic regions of genes scoring in the top 1%, 5%, and 10% in terms of up- and downregulation. The number of genomic regions analyzed is indicated below each plot.

Since the Dnmts regulate the expression of a number of genes, we next examined the relationship between replication timing changes and transcriptional changes. We performed genome-wide gene expression analysis in DKO cells with and without Dnmt3a2 induction. As expected, several genes within the *MageA* and *Rhox* gene clusters known to be regulated by DNA methylation [18,49] were downregulated upon Dnmt3a2 induction (data not shown). In addition, a subset of genes was upregulated, which might have been partly due to the indirect effects of Dnmt3a2 induction. We found that EtoL switching regions upon Dnmt3a2 induction tended to harbor downregulated genes (Figure 4A,B). In LtoE switching regions, coordinate changes in replication and transcription were not evident when taking all the expressed genes within these regions into account (Figure 4B). However, a subset of highly upregulated (>two-fold) genes was found to be enriched in the LtoE switching regions (Figure S7). This led us to focus on the genes that were highly up- and downregulated upon Dnmt3a2 induction to examine their relationship with replication timing changes (Figure 4C). Since most of the highly up- and downregulated genes were not localized in the EtoL and LtoE switching regions (Figure S7), we extended the analysis to the chromosomal regions harboring the genes scoring in the top 1%, 5%, and 10% in terms of up- and downregulation, regardless of their statistical significance in replication timing switching. Interestingly, we found that highly up- and downregulated transcription was associated with shifts in replication timing towards earlier and later timing, respectively. A similar but less pronounced result was obtained from the comparison between WT and TKO (Figure S8). Collectively, these results suggest that transcriptional changes, either directly or indirectly induced by Dnmt loss, could be a cause or consequence of replication timing changes in the chromosomal arm regions.

## 4. Discussion

Using Dnmt-deficient mouse ES cells as a model, our study uncovered widespread effects of Dnmt loss on the mammalian DNA replication landscape and also highlighted the complexity of the mechanisms behind induced changes in replication timing.

Within PH regions, loss of DNA methylation destabilizes constitutive heterochromatin structure, leading to regional chromatin reorganization that involves abnormal enrichment of H3K27me3 foci. Loss of DNA methylation along CG-rich sequences is known to allow targeting of polycomb protein KDM2B, which promotes the formation of the H3K27me3 mark via PRC2 recruitment [50,51], and this is likely also the case for H3K27me3 foci formation within the PH of TKO cells. Consistent with this, we observed enrichment of polycomb group proteins at H3K27me3 foci (data not shown). Because H3K27me3-enriched regions replicate later than neighboring PH regions where constitutive heterochromatin marks are normally maintained without DNA methylation, we concluded that while the loss of DNA methylation is necessary, it is not alone responsible for the later replication of PH—chromatin reorganization is also required. Although we do not exclude the possibility that H3K27me3 affects the temporal order of PH replication, it is also possible that either gain or loss of other epigenetic modifications is involved in this phenomenon. For example, Suv4-20h-mediated H4K20me3 has been shown to be necessary for origin activation in a certain genome context [52], and we observed loss of this modification at H3K27me3 foci (Figure S2C,D).

A previous study has shown that partial loss of DNA methylation within PH advances replication timing [53]. For example, *Dnmt1* KO ES cells in which 30% of the methylation is still maintained show earlier replicating major satellite sequences than control wild-type ES cells [53]. Further extensive loss of DNA methylation induces H3K27me3 foci formation within PH and may shift this region back towards late replication. In other words, replication timing of PH may fluctuate depending on the DNA methylation level. It should be noted that H3K27me3 foci are not formed in *Dnmt1* single KO cells (data not shown). Abnormal enrichment of the H3K27me3 mark was also observed in histone H3K9 methyltransferase Suv39h1/h2 double-knockout ES cells [54], and delayed replication timing of PH was induced in these cells [53]. Although these lines of evidence suggest the close relationship between the H3K27me3 mark and replication timing regulation, the detailed mechanistic link behind this relationship is currently unclear.

Interestingly, the relationship between H3K27me3 and the replication timing changes discussed above seems to be restricted to PH. Our genome-wide analysis revealed that altered enrichment of the H3K27me3 mark in TKO ES cells is largely uncorrelated with changes in replication timing, suggesting that the mechanism by which the loss of DNA methylation affects the temporal order of PH replication is not involved in the replication timing changes seen in the chromosomal arm regions. Thus, multiple mechanisms are thought to be involved in the observed replication timing changes in Dnmt-deficient ES cells.

We showed that earlier and later shifts in replication timing are often associated with transcriptional up- and downregulation, respectively. As this trend is evident when major changes in transcription occur in response to Dnmt3a2 reintroduction into DKO ES cells, transcription itself or the associated chromatin changes could be a driving force behind the replication timing changes. However, we do not exclude the possibility that replication timing changes affect transcriptional activity. It has been shown that plasmid DNA injected into early and late S phase cell nuclei preferentially forms active and inactive chromatin structures, respectively, via an as yet unknown mechanism [55]. This replication timing-dependent assembly of different types of chromatin could have an impact on gene transcription. The relationship between transcription and DNA replication could, thus, be interdependent through a positive feedback loop, making it difficult to determine which is the cause and which the consequence. Coordinate changes between replication timing and transcription are also observed during mouse ES cell differentiation [4,5]. Genes in EtoL and LtoE switching regions are often down- and upregulated, respectively. Analysis at multiple intermediate differentiation stages has revealed no consistent relationship between transcription and replication that definitively illustrated which change is an upstream event: Some genes undergo transcriptional changes prior to replication timing switching, whereas others undergo transcriptional changes that follow replication timing switching [5].

Considering that significant changes in epigenetic modifications and gene transcription are induced in Dnmt-deficient cells, it may be surprising that the observed changes in replication timing are relatively subtle and that ES cell-specific Mb-sized replication domain organization is globally maintained. However, this was supported by recent electron spectroscopic and Hi-C experimental findings that higher-ordered chromatin structures and A/B compartment structures are largely maintained in the absence of the Dnmts [56,57]. The observed robustness of the replication domain structure could be partly explained by the replication timing program being regulated at two different levels (i.e., at the replication domain level and the replication origin level) in mammalian cells [3,7]. Unlike in yeast, replication timing in mammalian cells is first determined at the level of Mb-sized replication domains during the early G1 phase, which determines the domains to be replicated during the early or late S phase. In the subsequent step during the G1 phase, replication origin selection occurs to determine the local order of replication within each individual (early or late) domain. If regional changes in chromatin structure or transcriptional activity that affect local origin activity occur in response to Dnmt loss, the effects would tend to be buffered by the replication forks of neighboring origins within the same domain (the distances between adjacent origins are estimated to range between 40–150 kb based on DNA fiber experiments), resulting in the robustness of Mb-sized replication domain organization.

## 5. Conclusions

In summary, our study elucidated the extent to which DNA methylation and the Dnmts contribute to replication timing regulation. This will provide a basis for understanding diseases associated with chromatin states and replication timing dysregulation.

## Data Availability

Microarray and next generation sequencing data obtained in this study were deposited in the Gene Expression Omnibus (GEO) database under the accession number, GSE119479.

## Supplementary Materials

The following are available online at https://www.mdpi.com/2073-4409/10/2/266/s1, Figure S1: DNA replication fork progression rate and ori-to-ori distances as measured in Dnmt-depleted cells, Figure S2: Extensive loss of DNA methylation results in the formation of H3K27me3 foci within pericentromeric major satellite repeats, Figure S3: H3K27me3 foci are formed in a DNA methylation-dependent manner, Figure S4: Establishment of a DKO ES cell clone stably expressing RU486-inducible wild-type Dnmt3a2, Figure S5: Reproducibility of the replication timing changes upon Dnmt depletion, Figure S6: Pluripotent cell-specific replication timing is retained in Dnmt-depleted ES cells, Figure S7: Summary of the number of genes showing more than two-fold change within the LtoE, EtoL, EtoE, and LtoL regions defined in Figure 4A, Figure S8: Relationship between replication timing changes and transcription changes in TKO cells (the same analysis done in Figure 4C).

## References

[1] Berezney R., Dubey D.D., Huberman J.A. (2000). Heterogeneity of eukaryotic replicons, replicon clusters, and replication foci. Chromosoma.

[2] Pope B.D., Gilbert D.M. (2013). The replication domain model: Regulating replicon firing in the context of large-scale chromosome architecture. J. Mol. Biol..

[3] Takebayashi S.-I., Ogata M., Okumura K. (2017). Anatomy of mammalian replication domains. Genes.

[4] Hiratani I., Ryba T., Itoh M., Yokochi T., Schwaiger M., Chang C.-W., Lyou Y., Townes T.M., Schübeler D., Gilbert D.M. (2008). Global reorganization of replication domains during embryonic stem cell differentiation. PLoS Biol..

[5] Hiratani I., Ryba T., Itoh M., Rathjen J., Kulik M., Papp B., Fussner E., Bazett-Jones D.P., Plath K., Dalton S. (2009). Genome-wide dynamics of replication timing revealed by in vitro models of mouse embryogenesis. Genome Res..

[6] Ryba T., Battaglia D., Chang B.H., Shirley J.W., Buckley Q., Pope B.D., Devidas M., Druker B.J., Gilbert D.M. (2012). Abnormal developmental control of replication-timing domains in pediatric acute lymphoblastic leukemia. Genome Res..

[7] Dileep V., Rivera-Mulia J.C., Sima J., Gilbert D.M. (2015). Large-scale chromatin structure–function relationships during the cell cycle and development: Insights from replication timing. Cold Spring Harb. Symp. Quant. Biol..

[8] Bird A. (2002). DNA methylation patterns and epigenetic memory. Genes Dev..

[9] Bernstein B.E., Meissner A., Lander E.S. (2007). The mammalian epigenome. Cell.

[10] Jones P.A., Liang G. (2009). Rethinking how DNA methylation patterns are maintained. Nat. Rev. Genet..

[11] Law J.A., Jacobsen S.E. (2010). Establishing, maintaining and modifying DNA methylation patterns in plants and animals. Nat. Rev. Genet..

[12] Zhu H., Wang G., Qian J. (2016). Transcription factors as readers and effectors of DNA methylation. Nat. Rev. Genet..

[13] Meehan R.R., Lewis J.D., McKay S., Kleiner E.L., Bird A.P. (1989). Identification of a mammalian protein that binds specifically to DNA containing methylated CpGs. Cell.

[14] Leonhardt H., Page A.W., Weier H.-U., Bestor T.H. (1992). A targeting sequence directs DNA methyltransferase to sites of DNA replication in mammalian nuclei. Cell.

[15] Bachman K.E., Rountree M.R., Baylin S.B. (2001). Dnmt3a and Dnmt3b are transcriptional repressors that exhibit unique localization properties to heterochromatin. J. Biol. Chem..

[16] Okano M., Bell D.W., Haber D.A., Li E. (1999). DNA methyltransferases Dnmt3a and Dnmt3b are essential for de novo methylation and mammalian development. Cell.

[17] Jackson-Grusby L., Beard C., Possemato R., Tudor M., Fambrough D., Csankovszki G., Dausman J., Lee P., Wilson C., Lander E. (2001). Loss of genomic methylation causes p53-dependent apoptosis and epigenetic deregulation. Nat. Genet..

[18] Oda M., Yamagiwa A., Yamamoto S., Nakayama T., Tsumura A., Sasaki H., Nakao K., Li E., Okano M. (2006). DNA methylation regulates long-range gene silencing of an X-linked homeobox gene cluster in a lineage-specific manner. Genes Dev..

[19] Denis H., Ndlovu M.N., Fuks F. (2011). Regulation of mammalian DNA methyltransferases: A route to new mechanisms. EMBO Rep..

[20] Rose N.R., Klose R.J. (2014). Understanding the relationship between DNA methylation and histone lysine methylation. Biochim. Biophys. Acta Bioenerg..

[21] Eden A., Gaudet F., Waghmare A., Jaenisch R. (2003). Chromosomal instability and tumors promoted by DNA hypomethylation. Science.

[22] Robertson K.D. (2005). DNA methylation and human disease. Nat. Rev. Genet..

[23] Tuck-Muller C.M., Narayan A., Tsien F., Smeets D.F., Sawyer J., Fiala E.S., Sohn O.S., Ehrlich M. (2000). DNA hypomethylation and unusual chromosome instability in cell lines from ICF syndrome patients. Cytogenet. Cell Genet..

[24] Dodge J.E., Okano M., Dick F., Tsujimoto N., Chen T., Wang S., Ueda Y., Dyson N., Li E. (2005). Inactivation of Dnmt3b in mouse embryonic fibroblasts results in DNA hypomethylation, chromosomal instability, and spontaneous immortalization. J. Biol. Chem..

[25] Rodriguez J., Frigola J., Vendrell E., Risques R.-A., Fraga M.F., Morales C., Moreno V., Esteller M., Capellà G., Ribas M. (2006). Chromosomal instability correlates with genome-wide DNA demethylation in human primary colorectal cancers. Cancer Res..

[26] Suzuki M., Oda M., Ramos M.-P., Pascual M., Lau K., Stasiek E., Agyiri F., Thompson R.F., Glass J.L., Jing Q. (2011). Late-replicating heterochromatin is characterized by decreased cytosine methylation in the human genome. Genome Res..

[27] Tsumura A., Hayakawa T., Kumaki Y., Takebayashi S.-I., Sakaue M., Matsuoka C., Shimotohno K., Ishikawa F., Li E., Ueda H.R. (2006). Maintenance of self-renewal ability of mouse embryonic stem cells in the absence of DNA methyltransferases Dnmt1, Dnmt3a and Dnmt3b. Genes Cells.

[28] Wan Y., Coxe K.K., Thackray V.G., Housley P.R., Nordeen S.K. (2001). Separable features of the ligand-binding domain determine the differential subcellular localization and ligand-binding specificity of glucocorticoid receptor and progesterone receptor. Mol. Endocrinol..

[29] Vegeto E., Allan G.F., Schrader W.T., Tsai M.-J., McDonnell D.P., O’Malley B.W. (1992). The mechanism of RU486 antagonism is dependent on the conformation of the carboxy-terminal tail of the human progesterone receptor. Cell.

[30] Wang Y., Xu J., Pierson T., O’Malley B.W., Tsai S.Y. (1997). Positive and negative regulation of gene expression in eukaryotic cells with an inducible transcriptional regulator. Gene Ther..

[31] Ryba T., Battaglia D., Pope B.D., Hiratani I., Gilbert D.M. (2011). Genome-scale analysis of replication timing: From bench to bioinformatics. Nat. Protoc..

[32] Takebayashi S.-I., Lei I., Ryba T., Sasaki T., Dileep V., Battaglia D., Gao X., Fang P., Fan Y., Esteban M.A. (2013). Murine esBAF chromatin remodeling complex subunits BAF250a and Brg1 are necessary to maintain and reprogram pluripotency-specific replication timing of select replication domains. Epigenet. Chromatin.

[33] Takebayashi S.-I., Ogata S., Ogata M., Okumura K. (2018). Mapping mammalian replication domains using the ion torrent semiconductor sequencing platform. Biosci. Biotechnol. Biochem..

[34] Ryba T., Hiratani I., Sasaki T., Battaglia D., Kulik M., Zhang J., Dalton S., Gilbert D.M. (2011). Replication timing: A fingerprint for cell identity and pluripotency. PLoS Comput. Biol..

[35] Kuriya K., Higashiyama E., Avşar-Ban E., Tamaru Y., Ogata S., Takebayashi S.-I., Ogata M., Okumura K. (2015). Direct visualization of dna replication dynamics in zebrafish cells. Zebrafish.

[36] Michalet X., Ekong R., Fougerousse F., Rousseaux S., Schurra C., Hornigold N., van Slegtenhorst M., Wolfe J., Povey S., Beckmann J.S. (1997). Dynamic molecular combing: Stretching the whole human genome for high-resolution studies. Science.

[37] Koberna K., Staněk D., Malínský J., Eltsov M., Pliss A., Čtrnáctá V., Cermanová Š., Raška I. (1999). Nuclear organization studied with the help of a hypotonic shift: Its use permits hydrophilic molecules to enter into living cells. Chromosoma.

[38] Hino S., Sakamoto A., Nagaoka K., Anan K., Wang Y., Mimasu S., Umehara T., Yokoyama S., Kosai K.-I., Nakao M. (2012). FAD-dependent lysine-specific demethylase-1 regulates cellular energy expenditure. Nat. Commun..

[39] Takebayashi S.-I., Tamura T., Matsuoka C., Okano M. (2007). Major and essential role for the DNA methylation mark in mouse embryogenesis and stable association of DNMT1 with newly replicated regions. Mol. Cell. Biol..

[40] Panning M.M., Gilbert D.M. (2005). Spatio-temporal organization of DNA replication in murine embryonic stem, primary, and immortalized cells. J. Cell. Biochem..

[41] Takebayashi S.-I., Sugimura K., Saito T., Sato C., Fukushima Y., Taguchi H., Okumura K. (2005). Regulation of replication at the R/G chromosomal band boundary and pericentromeric heterochromatin of mammalian cells. Exp. Cell Res..

[42] Cooper S., Dienstbier M., Hassan R., Schermelleh L., Sharif J., Blackledge N.P., De Marco V., Elderkin S., Koseki H., Klose R. (2014). Targeting polycomb to pericentric heterochromatin in embryonic stem cells reveals a role for H2AK119u1 in PRC2 recruitment. Cell Rep..

[43] Chang B.H., Smith L., Huang J., Thayer M. (2006). Chromosomes with delayed replication timing lead to checkpoint activation, delayed recruitment of Aurora B and chromosome instability. Oncogene.

[44] Cornacchia D., Dileep V., Quivy J.-P., Foti R., Tili F., Santarella-Mellwig R., Antony C., Almouzni G., Gilbert D.M., Buonomo S.B.C. (2012). Mouse Rif1 is a key regulator of the replication-timing programme in mammalian cells. EMBO J..

[45] Yamazaki S., Ishii A., Kanoh Y., Oda M., Nishito Y., Masai H. (2012). Rif1 regulates the replication timing domains on the human genome. EMBO J..

[46] Fernandez-Vidal A., Guitton-Sert L., Cadoret J.-C., Drac M., Schwob E., Baldacci G., Cazaux C., Hoffmann J.-S. (2014). A role for DNA polymerase θ in the timing of DNA replication. Nat. Commun..

[47] Reddington J.P., Perricone S.M., Nestor C.E., Reichmann J.A., Youngson N.A., Suzuki M., Reinhardt D., Dunican D.S., Prendergast J.G.D., Mjoseng H.K. (2013). Redistribution of H3K27me3 upon DNA hypomethylation results in de-repression of Polycomb target genes. Genome Biol..

[48] King A.D., Huang K., Rubbi L., Liu S., Wang C.-Y., Wang Y., Pellegrini M., Fan G. (2016). Reversible Regulation of promoter and enhancer histone landscape by DNA methylation in mouse embryonic stem cells. Cell Rep..

[49] Karimi M.M., Goyal P., Maksakova I.A., Bilenky M., Leung D., Tang J.X., Shinkai Y., Mager D.L., Jones S., Hirst M. (2011). DNA Methylation and SETDB1/H3K9me3 regulate predominantly distinct sets of genes, retroelements, and chimeric transcripts in mESCs. Cell Stem Cell.

[50] Farcas A.M., Blackledge N.P., Sudbery I., Long H.K., McGouran J.F., Rose N.R., Lee S., Sims D., Cerase A., Sheahan T.W. (2012). KDM2B links the polycomb repressive complex 1 (PRC1) to recognition of CpG islands. elife.

[51] Wu X., Johansen J.V., Helin K. (2013). Fbxl10/Kdm2b recruits polycomb repressive complex 1 to CpG islands and regulates H2A ubiquitylation. Mol. Cell.

[52] Brustel J., Kirstein N., Izard F., Grimaud C., Prorok P., Cayrou C., Schotta G., Abdelsamie A.F., Déjardin J., Méchali M. (2017). Histone H4K20 tri-methylation at late-firing origins ensures timely heterochromatin replication. EMBO J..

[53] Jørgensen H.F., Azuara V., Amoils S., Spivakov M., Terry A., Nesterova T.B., Cobb B.S., Ramsahoye B., Merkenschlager M., Fisher A.G. (2007). The impact of chromatin modifiers on the timing of locus replication in mouse embryonic stem cells. Genome Biol..

[54] Peters A.H., Kubicek S., Mechtler K., O’Sullivan R.J., Derijck A.A., Perez-Burgos L., Kohlmaier A., Opravil S., Tachibana M., Shinkai Y. (2003). Partitioning and plasticity of repressive histone methylation states in mammalian chromatin. Mol. Cell.

[55] Zhang J., Xu F., Hashimshony T., Keshet I., Cedar H. (2002). Establishment of transcriptional competence in early and late S phase. Nature.

[56] Hassan-Zadeh V., Rugg-Gunn P., Bazett-Jones D.P. (2017). DNA methylation is dispensable for changes in global chromatin architecture but required for chromocentre formation in early stem cell differentiation. Chromosoma.

[57] Nothjunge S., Nührenberg T.G., Grüning B.A., Doppler S.A., Preissl S., Schwaderer M., Rommel C., Krane M., Hein L., Gilsbach R. (2017). DNA methylation signatures follow preformed chromatin compartments in cardiac myocytes. Nat. Commun..

