# Supplementary material for: The Temporal Order of DNA Replication Shaped by Mammalian DNA Methyltransferases
Source: Cells. 2021 Jan 29;10(2):266. doi: 10.3390/cells10020266 (PMC7911666; doi:10.3390/cells10020266)
Supplement: Supplementary file 1 [file cells-10-00266-s001.pdf]

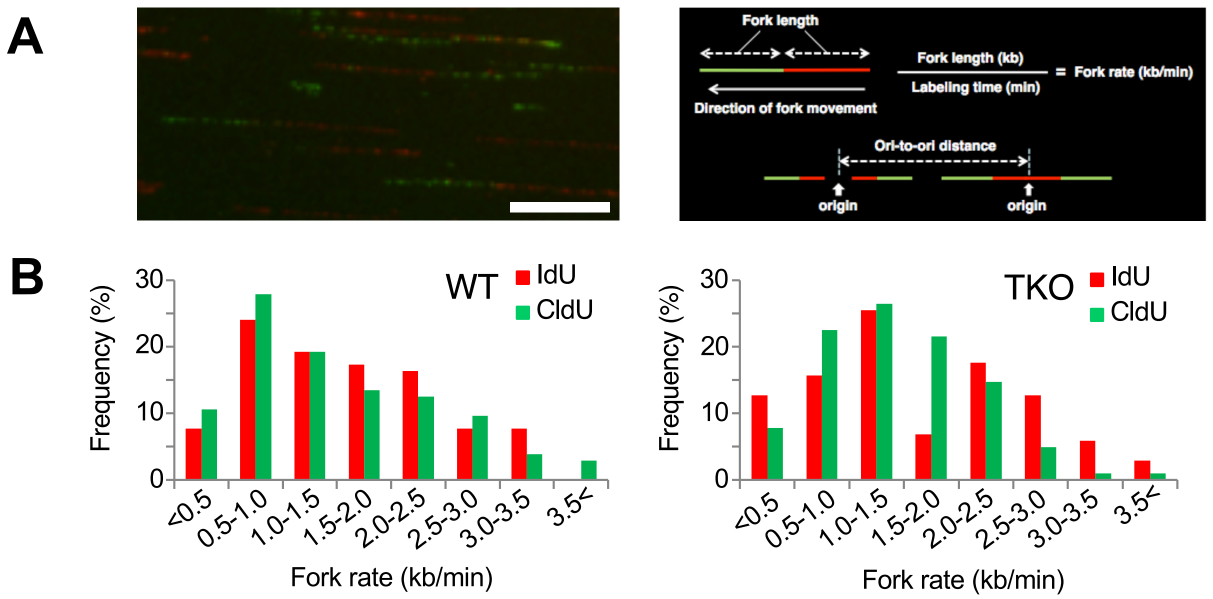

	WT IdU	WT CldU	TKO IdU	TKO CldU
Average (kb/min)	1.60	1.54	1.63	1.43
Median (kb/min)	1.47	1.25	1.44	1.36
S.D.	0.84	0.94	0.94	0.74
N	104	104	102	102

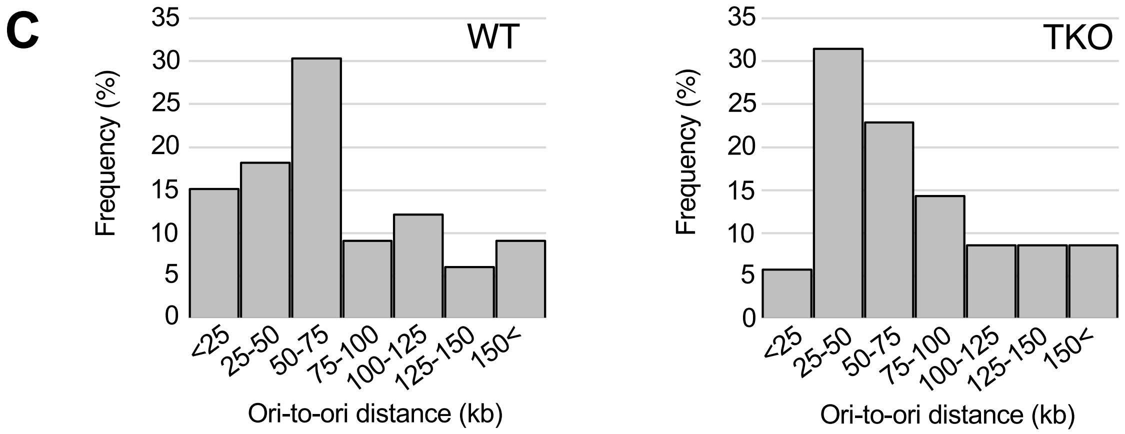

	WT	TKO
Average (kb)	77.8	74.9
Median (kb)	63.1	63.6
S.D.	60.6	43.6
N	33	35

Figure S1. DNA replication fork progression rate and ori-to-ori distances as measured in Dnmt-depleted cells. (**A**) A representative image of DNA replication fork progression in wild-type and TKO ES cells as visualized by consecutive pulse labeling with IdU for 20 min (red) and CldU for 20 min (green). Labeled DNA combed on the coverslip was detected by immunofluorescence staining. A conventional fluorescence microscope (Axioplan 2 MOT; Carl Zeiss) equipped with an ORCA R2 camera (Hamamatsu) was used for imaging of combed DNA. Scale bar, 20 kb. The rate of replication fork progression (kb/min) was calculated based on the length of the fork and the labeling time (see details in the Methods). (**B**) Histograms of analyzed replication fork rates in wild-type and extensively DNA hypomethylated TKO ES cells. N, number of analyzed forks. There was no statistically significant difference in the fork progression rates between the wild-type and TKO ES cells (IdU, $P = 0.95$; CldU, $P = 0.83$; Mann–Whitney U test). (**C**) Histograms of the measured ori-to-ori distances in wild-type and TKO ES cells. There was no statistically significant difference in the ori-to-ori distances between these cell lines ($P = 0.77$, Mann–Whitney U test). N, number of calculated ori-to-ori distances.

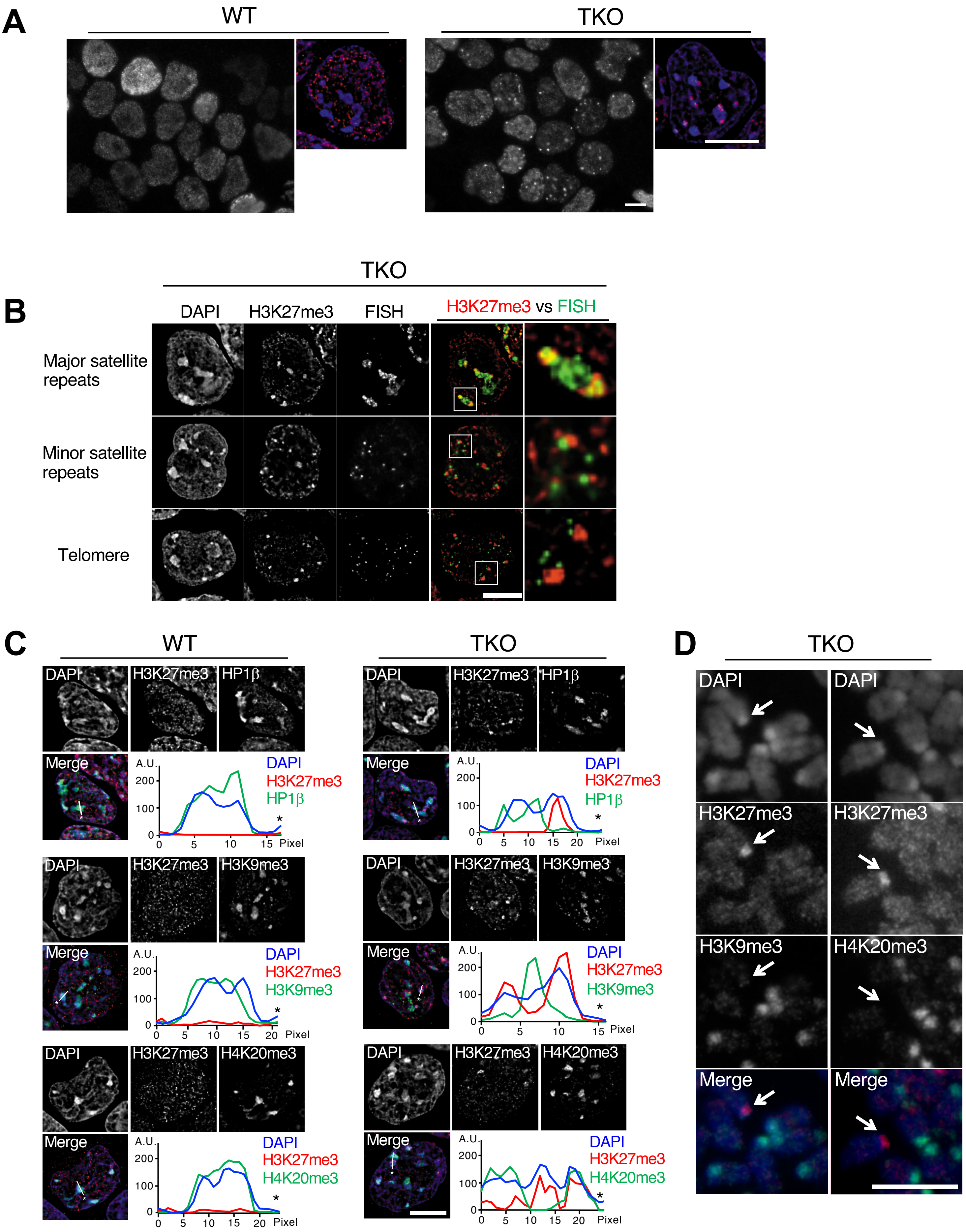
A
WT
TKO
B
TKO
DAPI
H3K27me3
FISH
H3K27me3 vs FISH
Major satellite repeats
Minor satellite repeats
Telomere
C
WT
TKO
DAPI
H3K27me3
HP1β
H3K9me3
H4K20me3
Merge
A.U.
Pixel
D
TKO
DAPI
H3K27me3
H3K9me3
H4K20me3
Merge

Figure S2. Extensive loss of DNA methylation results in the formation of H3K27me3 foci within pericentromeric major satellite repeats. (**A**) H3K27me3 foci are formed in extensively DNA hypomethylated TKO ES cells. H3K27 methylation signals are distributed throughout euchromatin in wild-type ES cells (left panel). In the extensively hypomethylated TKO ES cells, signals are abnormally enriched within discrete domains, termed H3K27me3 foci (right panel). Overlay with DAPI (blue) revealed that the H3K27 methylation foci (red) were formed within DAPI-dense pericentromeric heterochromatin (PH) regions. (**B**) H3K27 methylation foci were observed to form in the region of major satellite repeats. Major satellite repeats (top), minor satellite repeats (middle), and telomere repeats (bottom) were detected simultaneously with H3K27me3 foci in interphase nuclei by immunofluorescence in situ hybridization (immuno-FISH) by targeting specific repetitive sequences. Only the pericentromeric major satellite repeat signals (green) overlapped with the H3K27me3 foci (red), resulting in yellow signals in the merged images. (**C**) Regional loss of heterochromatin markers occurred at the H3K27me3 foci. Colocalization analysis between the H3K27me3 foci and several heterochromatin markers (HP1β, H3K9me3, and H4K20me3) in the PH of the wild-type (left) and TKO (right) ES cells. DAPI, H3K27me3, and the heterochromatin markers were imaged on the same focal plane and colored in blue, red, and green, respectively, in the merged images. The charts show the immunofluorescence signal intensities (A.U., arbitrary units) plotted along the white line shown in each of the magnified images of the PH. In wild-type ES cells, the distribution patterns of the heterochromatin markers (green lines) correlated well with those of DAPI (blue lines), indicating that these markers were enriched in the PH. The intensity of H3K27 methylation (red) was low in these cells. In the TKO ES cells, enrichment of heterochromatin markers was absent from the H3K27me3 foci. (**D**) The distribution patterns of the heterochromatin markers (H3K9me3 and H4K20me3) and H3K27me3 were mutually exclusive in the PH of mitotic chromosomes. Arrows indicate the H3K27me3-enriched PH of a mitotic chromosome showing depletion of the heterochromatin markers. The cell images were collected at multiple stage positions using a deconvolution fluorescence microscope (see details in **Materials and Methods**) and images at one focal plane are shown. Scale bars, 10 μm.

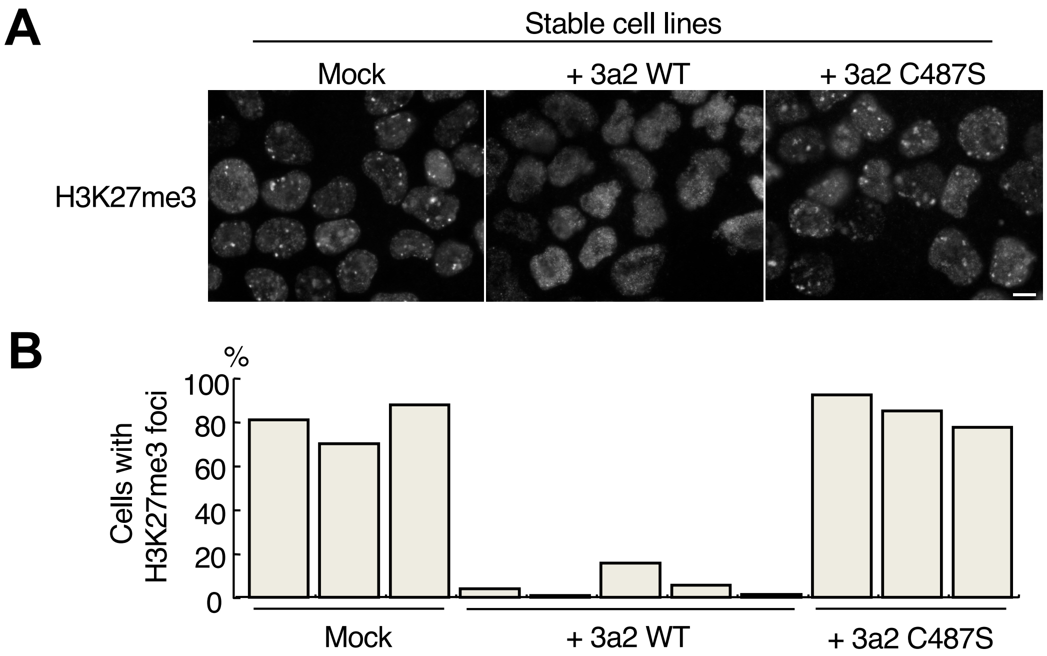

Figure S3. H3K27me3 foci are formed in a DNA methylation-dependent manner. (**A**) Immunostaining of H3K27me3 in DKO cell lines in which wild-type Dnmt3a2 or "catalytic-dead" Dnmt3a2 (C487S) was stably re-expressed. Cell lines containing an empty vector were used as the control (mock). The cell images were collected at multiple stage positions using a deconvolution fluorescence microscope (see details in **Materials and Methods**) and images at one focal plane are shown. Scale bar, 10 µm. (**B**) Percentage of cells with H3K27me3 foci in each cell line. H3K27 methylation foci formation was prevented only in those lines expressing wild-type Dnmt3a2 protein, suggesting that abnormal H3K27 foci are formed in a DNA methylation-dependent manner. Several independent cell clones were generated and used in this experiment.

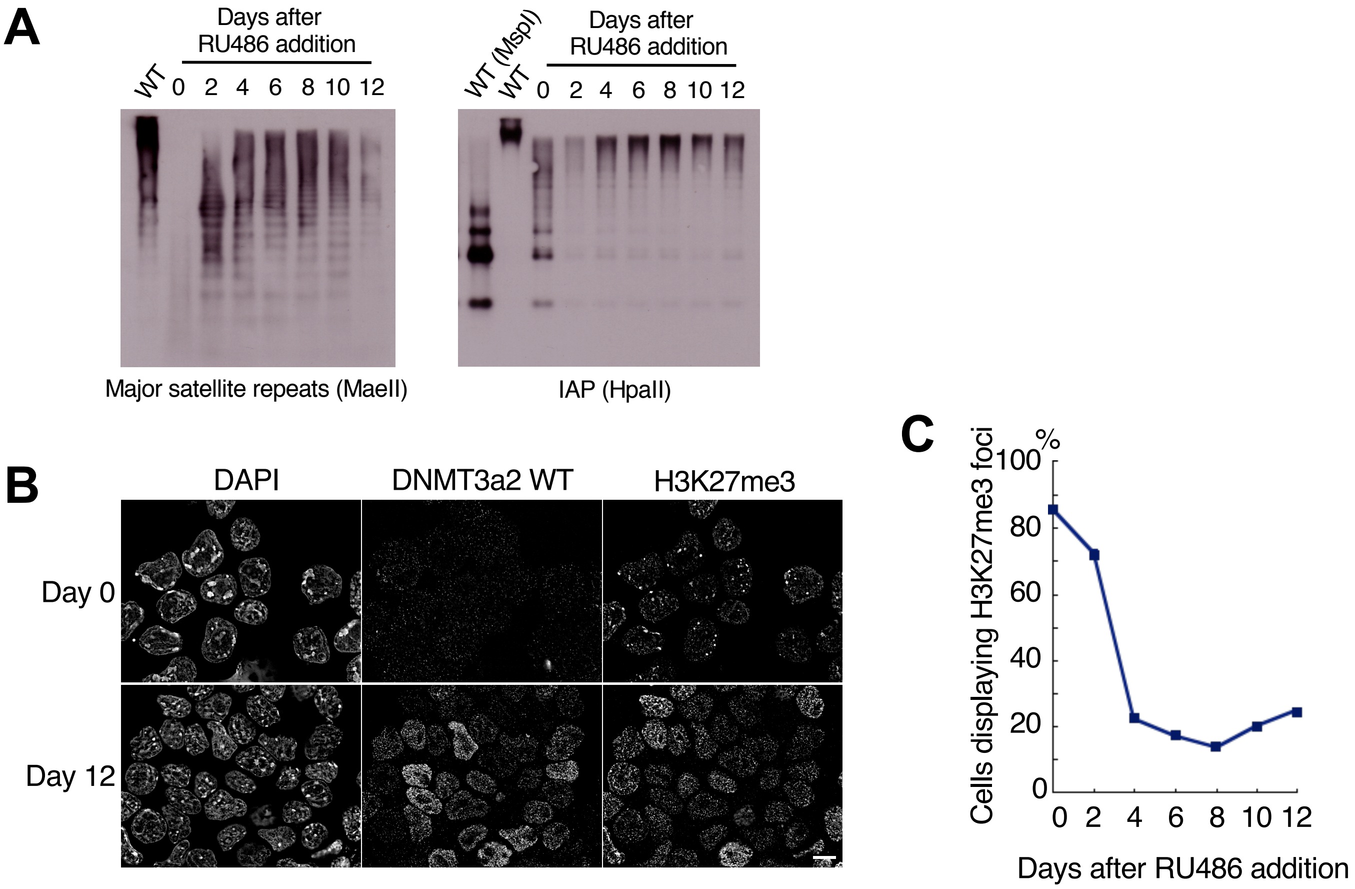

Figure S4. Establishment of a DKO ES cell clone stably expressing RU486-inducible wild-type Dnmt3a2. (**A**) DNA methylation of major satellite repeats and intracisternal A particle (IAP) was analyzed by Southern blotting in a *Dnmt3a*$^{-/-}$*Dnmt3b*$^{-/-}$ double-knockout (DKO) ES cell line stably expressing RU486-inducible Dnmt3a2. Genomic DNA was extracted from cells at each indicated time point after the addition of RU486 and then digested with methylation-sensitive (HpaII or MaeII) or methylation insensitive (MspI) restriction enzymes. Southern blotting was then carried out using the probe indicated at the bottom of each blot. DNA methylation in both the pericentromeric major satellite repeats and IAP was restored over time in the presence of RU486. (**B**) Immunostaining of Dnmt3a and H3K27me3 before (day 0) and after (day 12) RU486 treatment. The cell images were collected at multiple stage positions using a deconvolution fluorescence microscope (see details in **Materials and Methods**) and images at one focal plane are shown. Scale bar, 10 μm. (**C**) The percentage of cells with H3K27me3 foci was scored at each time point. Cells with intense H3K27me3 foci (apparently brighter than nucleoplasmic foci) at DAPI-dense pericentromeric heterochromatin regions were counted as foci-positive cells.

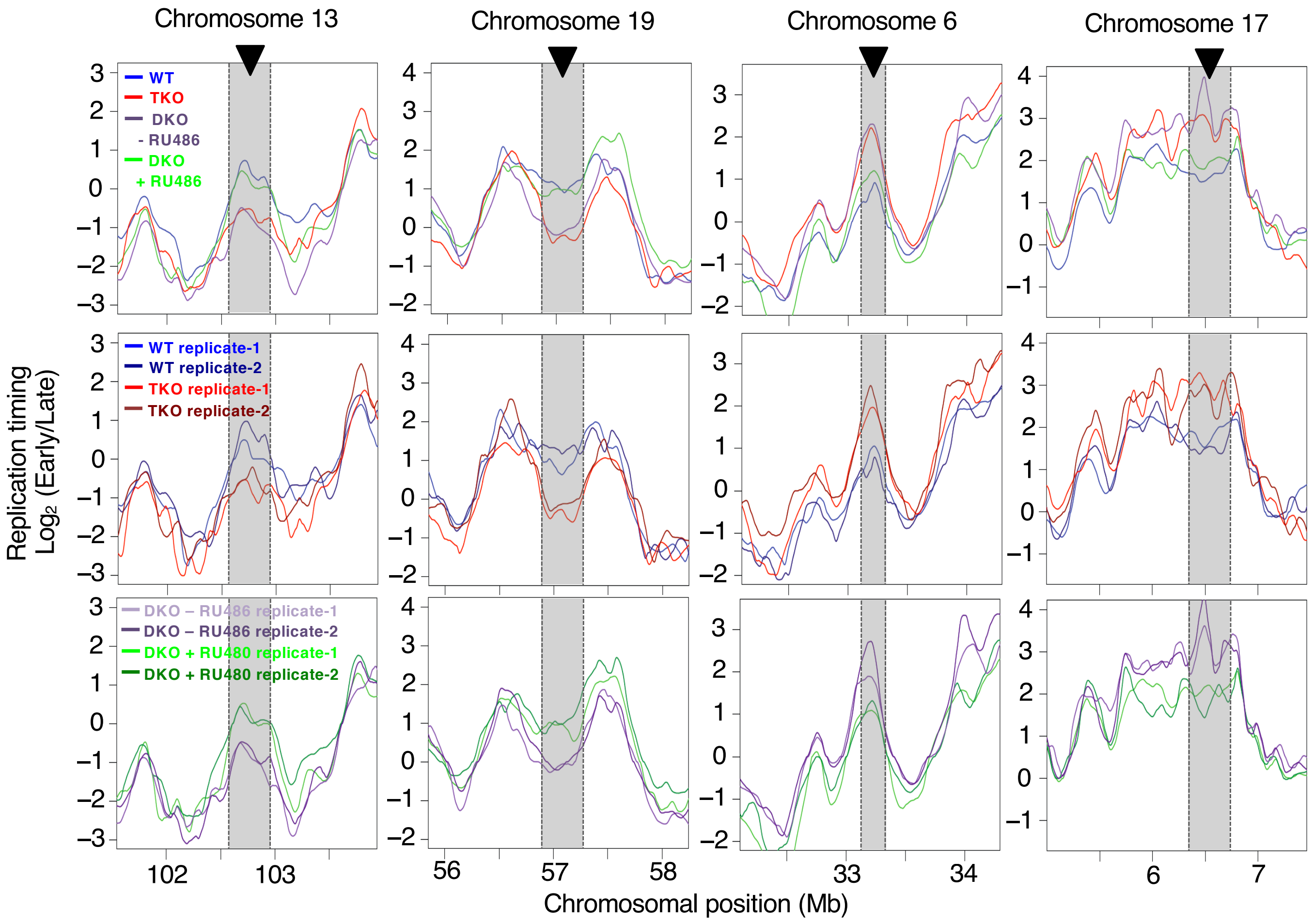

Figure S5. Reproducibility of the replication timing changes upon Dnmt depletion. The average replication timing profiles of representative timing switching regions from **Figure 2E** are shown in the top panels. Replication timing profiles of the same regions from two independent experiments are shown in the middle and bottom panels, indicating the high reproducibility of these timing changes.

A

	WT	TKO	DKO – RU486	DKO + RU486
**WT**	1.00	0.96	0.92	0.95
**TKO**	0.96	1.00	0.95	0.96
**DKO – RU486**	0.92	0.95	1.00	0.98
**DKO + RU486**	0.95	0.96	0.98	1.00
**46C (ES cell)**	0.92	0.93	0.92	0.93
**D3 (ES cell)**	0.89	0.92	0.92	0.92
**TT2 (ES cell)**	0.91	0.91	0.90	0.91
**NPC**	0.82	0.77	0.77	0.81
**Myo**	0.76	0.70	0.69	0.73
**MEF**	0.75	0.69	0.68	0.72

Differentiated cells (NPC, Myo, MEF)

Correlation (Pearson R)

B

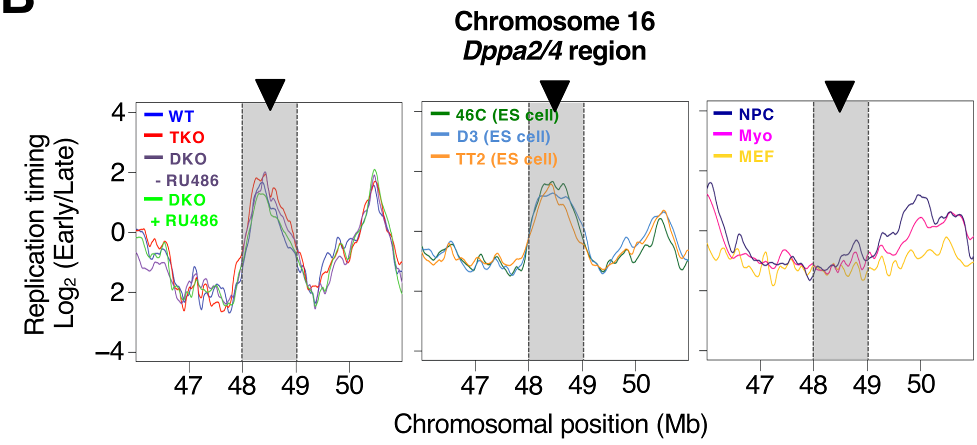

Figure S6. Pluripotent cell-specific replication timing is retained in Dnmt-depleted ES cells. (**A**) Correlation analysis (Pearson's r values) of the replication timing datasets from all the cell lines tested in this study. (**B**) Replication timing profiles of *Dppa2/4* regions from genome-wide analysis of each of the indicated cell lines.

Number of affected genes after DNMT3a2 induction (DKO + RU486 vs. DKO – RU486)

	>2 fold up	<2 fold down	Total number of genes within domains
LtoE (FDR = 1%)	7	0	579
LtoE (FDR = 5%)	16	2	1,880
EtoL (FDR = 1%)	0	0	125
EtoL (FDR = 5%)	2	5	404
EtoE (FDR = 10%)	47	33	9,260
LtoL (FDR = 10%)	39	14	1,868

Figure S7. Summary of the number of genes showing more than two-fold change within the LtoE, EtoL, EtoE, and LtoL regions defined in Figure 4A.

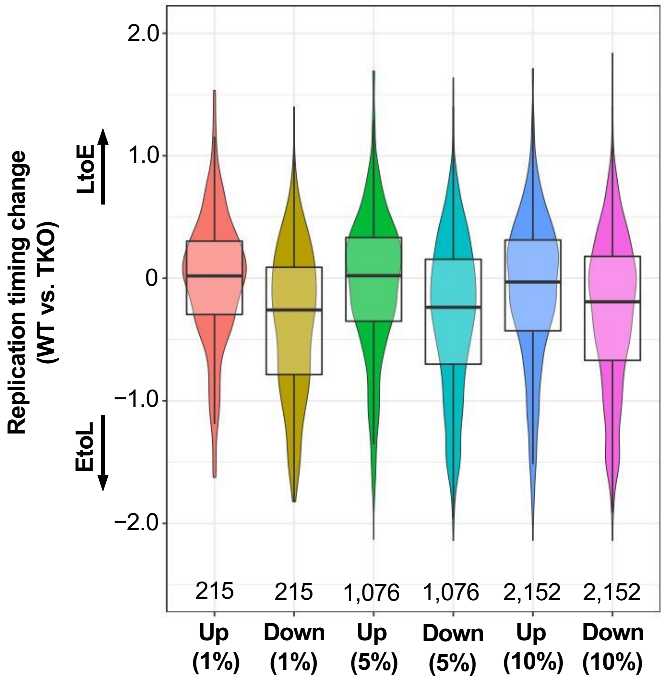

Figure S8. Relationship between replication timing changes and transcription changes in TKO cells (the same analysis done in Figure 4C). Box plots showing the replication timing changes in genomic regions of genes scoring in the top 1%, 5%, and 10% in terms of up- and down-regulation. The number of genomic regions analyzed is indicated below each plot. Gene expression data were obtained from the GEO database (accession number GSE20177).
